# sRNA23, a novel small RNA, regulates to the pathogenesis of *Streptococcus suis* serotype 2

**DOI:** 10.1080/21505594.2021.2008177

**Published:** 2021-12-09

**Authors:** Quanming Xu, Hong Chen, Wen Sun, Yongyi Zhang, Dewen Zhu, Kul Raj Rai, Ji-Long Chen, Ye Chen

**Affiliations:** a Fujian Agriculture and Forestry University, Fuzhou, China; b Key Laboratory of Fujian- Fujian Agriculture and Forestry University, Fuzhou, China

**Keywords:** *Streptococcus suis* serotype 2, RNA-seq, sRNA23, small RNA, pathogenesis

## Abstract

*Streptococcus suis* serotype 2 (*S. suis* 2) is an important ubiquitous zoonotic pathogen. To date, regulatory factors and their implication in *S. suis* pathogenesis are not fully understood. Small non-coding RNAs (sRNAs) have been proven to function as important regulatory factors in bacterial pathogenesis and stress adaptation. Here, we identified a differentially downregulated *S. suis* 05ZYH33 sRNA after iron starvation by RNA-seq, which we named sRNA23. The presence of sRNA23 was further confirmed by RACE and Northern blot. Expression of sRNA23 was significantly altered under different environmental stresses such as nutritional starvation, osmotic pressure, oxidative stress, and lysozymal exposure. A sRNA23-deleted mutant exhibited relatively shorter streptococcal chains and weakened biofilm-forming ability. The mutation also resulted in decreased adherence of the *S. suis* 05ZYH33 to human laryngeal epidermoid carcinoma (HEp-2) cells, increased sensitivity to phagocytosis by RAW264.7 macrophages, and significantly reduced hemolytic activity. Furthermore, we observed that a sRNA23-deleted mutant had a low survival rate in pig whole blood and attenuated virulence in a mouse model. Moreover, based on RNA pull-down and electrophoretic mobility shift assay, we found that sRNA23 can directly bind to two proteins involved in adhesion and biofilm formation, namely, moonlighting protein FBA (fructose diphosphate aldolase) and rplB (50S ribosomal protein L2), respectively. Collectively, sRNA23 enhances *S. suis* 2 pathogenicity and the binding between sRNA23 and FBA/rplB might play an essential role in the adherence and biofilm-forming ability of *S. suis* 2.

**Abbreviation:**

sRNA: small noncoding RNA; FBA: fructose diphosphate aldolase; rplB: 50S ribosomal protein L2; RACE: rapid amplification of cDNA ends; EMSA: electrophoretic mobility shift assay; THB: Todd-Hewitt broth; FBS: fetal bovine serum; BIP: 2,2ʹ-Bipyridine

## Introduction

*Streptococcus suis* is an important zoonotic pathogen, which threatens the health of humans and animals seriously and causes huge economic losses to the global pig industry. Among the 29 serotypes of *S. suis*, serotype 2 is the most widely distributed and most pathogenic worldwide [1]. Over the past decade, infections caused by this pathogen are on the rise [2]. Especially, two large-scale outbreaks of human infections of *S. suis* 2 occurred in China in 1998 and 2005. Since then, *S. suis* attracted a great deal of attention from a public and scientific point of view.

The pathogenic mechanism of *S. suis* mainly includes adhesion and colonization, sepsis and proliferation, immune activation and septic shock, and invasion of the central nervous system leading to meningitis. During environmental stress at various infection stages, *S. suis* induces a robust expression of various genes to achieve successful adhesion, invasion, and multiplication [1]. Various adhesion-related factors such as suilysin [3], muramidase-released protein [4], SsPepO [5], and SsPI-1 [6] are essential for host invasion. The arginine deiminase system can improve the acidic environment by synthesizing ammonia, thereby protecting itself from acid damage from phagolysosomes [7]. By modifying the capsular polysaccharide, *S. suis* evades the host’s immune surveillance and impairs the activation of NK cells [8]. These studies indicate that virulence factors play an important role in *S. suis* pathogenesis [1]. However, the expression of virulence factors is modulated by regulatory factors/systems at the transcription or translation level. It has been reported that there are two-component regulatory systems, transcription regulators, and other signal molecules in *S. suis* used to sense the external environment to regulate gene expression [9]. In addition to these common regulators, sRNAs have been regarded as a new type of regulatory factor found in bacteria in recent years. In *Streptococcus pneumoniae, Escherichia coli, Brucella, Salmonella, Vibrio cholerae*, etc., sRNAs have been widely reported to participate in the regulation of pathogenicity [10].

sRNAs, usually 50–500 nt in length, are a type of RNAs widely present in the transcriptome of prokaryotes but do not encode proteins. sRNAs perform regulatory functions mainly in two ways. First, *cis*/*trans*-encoded sRNA binds to target mRNA through complementary base pairing, thereby regulating the translation of mRNA or affecting the stability. For example, the antitoxin of *Bacillus subtilis* cis-encoded sRNA RatA and txpA mRNA form base complementary pairing. This hybrid formation generates substrate for RNase III and degrades txpA mRNA, thereby inhibiting the synthesis of TxpA [11]. The *trans*-encoded sRNA IsrM targets to SopA and HilE mRNA to regulate the expression of SPI-1, thereby affecting *Salmonella* invasion of epithelial cells and replication in macrophages [12]. Second, sRNA can directly bind to specific proteins and alters their function [13,14]. For example, sRNAs such as CsrB/C bind to the transcription regulatory conserved protein called CsrA and sequester the CsrA protein away from its target mRNA, thereby, regulating the expression of downstream genes [15]. Pathogenic bacteria can quickly adapt to changes in the microenvironment through sRNA-mediated mRNA expression or protein activity, and sRNAs are indispensable for regulating metabolism and the expression of virulence-related genes during colonization and invasion. A total of 37 sRNAs have been previously identified in *S. suis*, but only a few sRNAs have been analyzed for their regulatory mechanisms, such as rss04 [16], rss06 [17], and sRNA34 [18]. Among them, rss04 can regulate the expression of transcriptional regulator CcpA and virulence factor LuxS to inhibit the synthesis of capsular polysaccharides, thereby promoting the adhesion and invasion of *S. suis* to mouse brain microvascular endothelial cells [16]. The deletion of sRNA34 significantly prolongs the cellular chain, remarkably impairs the ability to anti-phagocytosis, and attenuates pathogenicity in mouse [18]. These studies suggest that sRNAs play crucial role in the pathogenesis of *S. suis*. However, there are many regulatory sRNAs in *S. suis* yet to be identified, and their involvement in the pathogenic process needs to be elucidated.

In this study, we identified a novel sRNA, which we called sRNA23, expressed by the highly virulent *S. suis* 2 strain 05ZYH33 through prokaryotic strand-specific transcriptome sequencing. In order to understand the regulatory roles of the novel sRNA, a sRNA23 mutant strain (ΔsRNA23) was constructed and phenotypic analysis was performed. Finally, specific proteins bound to sRNA23 were characterized based on RNA pull-down, and verified by electrophoretic mobility shift assay (EMSA).

## Results

### sRNA23 is downregulated sRNA in response to iron starvation

**Figure 1.**
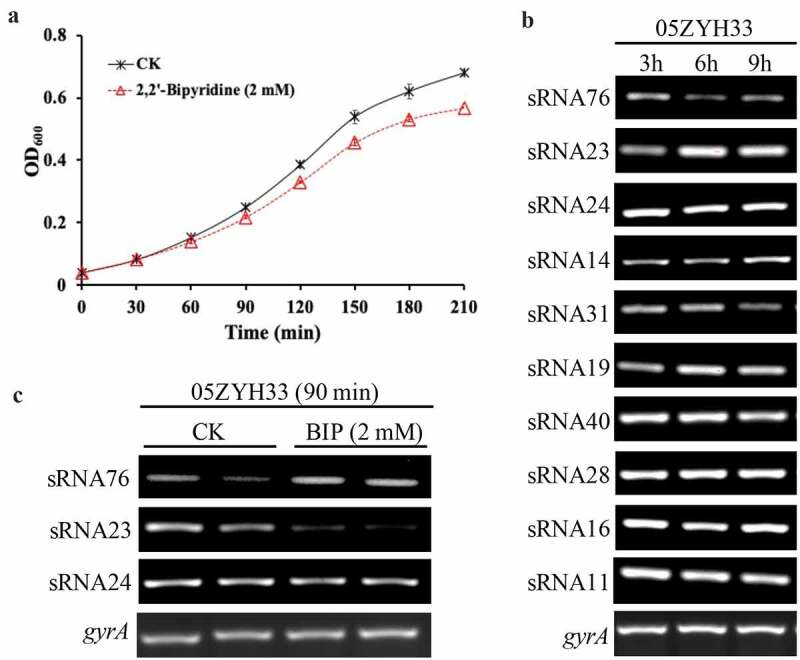
sRNA23 is a downregulated sRNA in response to iron starvation. (a) Growth of *S. suis* 05ZYH33 under iron starvation. *S. suis* were grown in THB + 5% FBS. After 30 minutes of bacterial culture, BIP was added to cultures. The black arrow indicates harvest time point for control and BIP-treated samples. (b) Expression levels of various sRNAs in *S. suis* 05ZYH33 at the indicated hours of post culture were examined by RT-PCR. (c) RT-PCR detection of three sRNAs of *S.suis* 05ZYH33 under iron starvation

*S. suis* 2 virulent strain, 05ZYH33, taken from a patient with streptococcal toxic shock syndrome [19], was used in this study. To study the differential gene expression profile in response to the iron starvation environment, the strain was cultured in THB with 5% fetal bovine serum (FBS) for 30 min (to the early exponential phase). 2,2ʹ-Bipyridine (BIP) was added to the medium to a final concentration of 2.5 mM to completely chelate the iron in the medium to create an iron-starved environment and then incubated for 60 min (Figure 1(a)). Total RNA was extracted and ribosomal RNA (rRNA) was removed, followed by a strand-specific library construction using deoxyuridine triphosphate (dUTP) second-strand marking method and then sequenced with the Illumina HiSeq 2000 system. The Rockhopper software was used to identify potential candidate sRNAs based on the 05ZYH33 reference genome annotations. A total of 14 different sRNAs were identified (Table S1) of which 10 were new sRNAs (previously uncharacterized). The length of the candidate sRNAs ranged from 100 to 500 nt. All candidate sRNAs were also detected successfully by RT-PCR in various growth phases such as lag, logarithmic, and stationery (Figure 1(b)). At the same time, RT-PCR was used to detect the expression of these 10 sRNAs under iron starvation and normal culture conditions. Expression trends of these sRNAs were consistent with RNA-seq. Iron starvation induced sRNA76 upregulation, while sRNA23 was significantly downregulated upon iron starvation. Moreover, sRNA24 was highly expressed in the early stages of bacterial growth phase (Figure 1(c)).

### sRNA23 deletion impairs the pathogenicity of 05ZYH33

**Figure 2.**
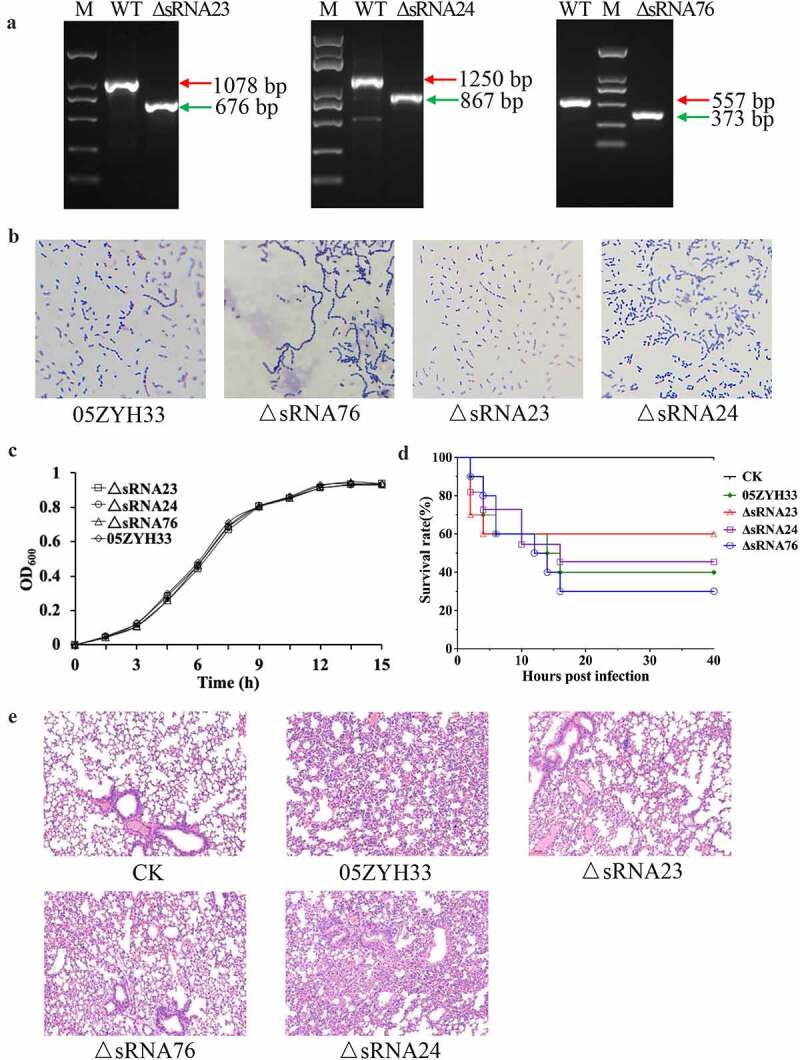
sRNA23 deletion impairs the pathogenicity of 05ZYH33. (a) Detection of mutant strains at the genome level by PCR using Out-F/R primers (Figure S1) respective to genomic DNA bands of WT and sRNA deleted mutants detected by gel electrophoresis. (b) Gram staining results of WT and *S. suis* mutants under light microscope (magnification, 1,000X). (c) Growth kinetics of 05ZYH33, ΔsRNA23, ΔsRNA24, and ΔsRNA76 cultured in THB + 5% FBS. (d) Survival rate of BALB/c mice infected with WT and mutant *S. suis*. BALB/c mice (5-week-old, female, ~22 g) were intraperitoneally injected with 0.4 mL (~5 × 10^8^ CFU) *S. suis* at the late-exponential growth phase cultured in THB + 5% FBS. (e) HE staining showing histopathological changes in mice infected with WT and mutant *S. suis* for 72 h. Images are representative of three independent experiments

It is reported that iron-responsive sRNAs not only regulate iron homeostasis, but also participate in the regulation of bacterial pathogenicity [20]. Because iron starvation caused a differential expression of sRNAs, we speculated that these differentially expressed sRNAs regulates *S. suis* 2 pathogenesis. Therefore, sRNA23, sRNA76, and sRNA24 deleted mutants were constructed by homologous recombination to study their functional role in *S. suis* 2 pathogenesis (Figure S1 and Figure 2(a)). We found that deletion of these sRNAs influenced the morphological changes of streptococcal-chain length of *S. suis* 2. Indeed, inactivation of sRNA76 caused elongation of the bacteria chain length, while sRNA23 inactivation shortened the chain length (Figure 2(b)). However, deletion of all these sRNAs had no significant effect on the growth pattern of 05ZYH33 (Figure 2(c)), indicating the deletion of these sRNAs may not influence bacterial growth under normal culture conditions. Previous studies showed that deletion of certain sRNA does not affect the bacterial growth under normal culture conditions but they are implicated in pathogenesis [21]. Next, a BALB/c mouse model was used to evaluate the effect of sRNA76, sRNA23 and sRNA24 deletion on the pathogenicity of *S. suis* 2. The survival rate of ΔsRNA76-infected mice was lower than that of the WT-infected group and ΔsRNA76-infected mice showed more severe alveolar wall swelling (Figure 2(d,e)). In contrast, the survival rate of ΔsRNA23-infected mice was higher than that of the WT strain infection group, and ΔsRNA23-infected mice had milder alveolar wall enlargement and congestion. The survival rate and pathological changes of ΔsRNA24-infected mice were comparable to the WT-infected group (Figure 2(d,e)). These results indicate that deletion of sRNA23 might impair the pathogenicity of *S. suis* 2 in mice. Therefore, sRNA23 was selected for further study to uncover its implication in the pathogenesis of *S. suis* 2.

### Genomic location and predicted structure of sRNA23

**Figure 3.**
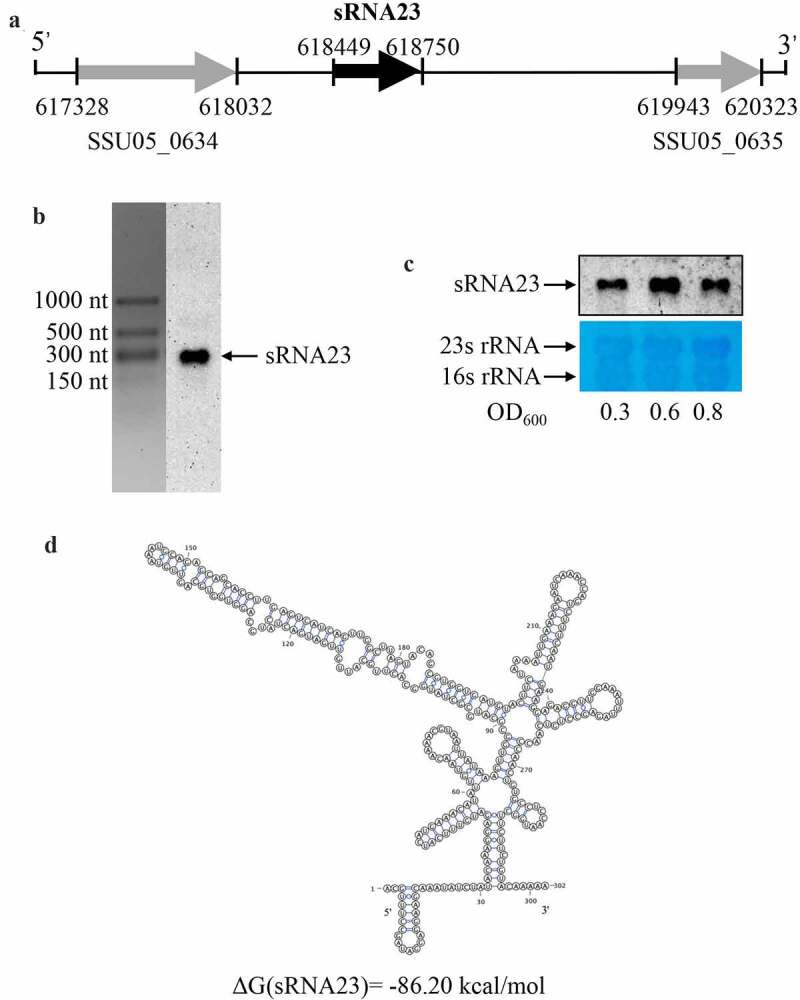
Genomic location and predicted structure of sRNA23. (a) The genomic position of sRNA 23 in *S. suis* 05ZYH33 was assessed by 5ʹ RACE and 3ʹ RACE. (b) Northern blot analysis for sRNA 23 of 05ZYH33 grown in THB + 5% FBS at exponential phase on formaldehyde denaturing 1.2% agarose gel. Dig-labeled RNA probes were used to detect target sRNA. The RNA marker was cut off and stained by GelStain. (c) Northern blot analysis of sRNA 23 expression at different growth phases (early-log (OD_600_: ~0.3), mid-log (OD_600_: ~0.6), and late-log (OD_600_: ~0.8)) of 05ZYH33. 23s rRNA and 16s rRNA were detected as loading controls by using methyl blue staining. (d) Predicted secondary structure of sRNA 23 by RNAfold

Firstly, the transcription start and termination sites of sRNA23 were determined using rapid amplification of 5ʹ-cDNA and 3ʹ-cDNA ends (RACE) analysis. We found that the transcription start and termination sites were located at positions 618,449 and 618,750, respectively (Figure 3(a), Table S2). sRNA23 is located in an intergenic region between two open reading frames: SSU05_0634 (Glucosamine-6-phosphate deaminase) and SSU05_0635 (Hypothetical protein) (Figure 3(a)). Next, Northern blot was performed to confirm the abundance and size of sRNA23 expression. The size (302 nt) of sRNA23 observed by Northern blot was consistent with that determined by RACE (Figure 3(b)). In addition, levels of sRNA23 expression at the early logarithmic phase to the late logarithmic phase were also determined, where a high level sRNA23 expression was found at the middle of the logarithmic phase (Figure 3(c)). Then, we predicted the secondary structure of sRNA23 by RNAfold (The Vienna RNA Web Services, http://rna.tbi.univie.ac.at) and illustrated it using VARNA GUI [22] (Figure 3(d)). BLASTn analysis suggested that sRNA23 is conserved among the 25 genomes of *S. suis* (serotypes 2, 14 and 1/2) but not in other *Streptococci* species in NCBI database (data not shown).

### sRNA23 is differentially expressed in a dose-dependent manner under different stress conditions

**Figure 4.**
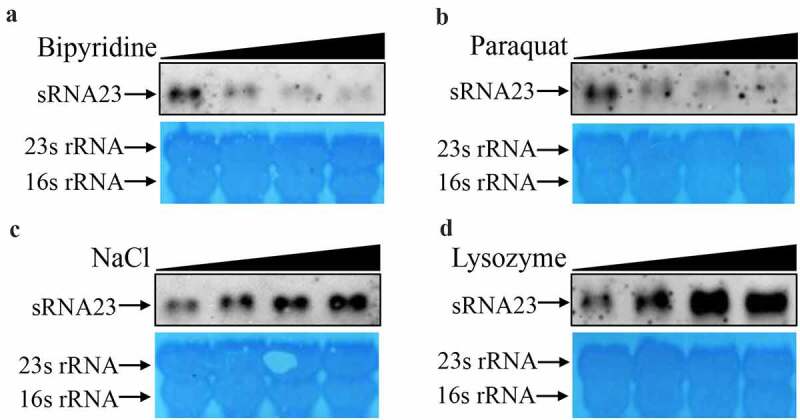
sRNA23 is differentially expressed in a dose-dependent manner under different stress conditions. Northern blot analysis of sRNA23 in *S. suis* 05ZYH33 strain after treating with different concentrations of (a) bipyridine (0, 1, 2, 4 mM), (b) paraquat (0, 2.5, 5.0, 10 mM), (c) NaCl (0,1, 2, 4 mM) and (d) lysozyme (0, 0.25, 0.5, 1 mg/mL)

To survive and thrive in a hostile environment (for example, the intestine, blood, and phagosomes), pathogens can quickly modulate the expression of various genes [23]. Recent reports showed that differential expression of sRNAs exhibited by pathogens plays critical role to adapt to the hostile microenvironment, and to regulate metabolism and pathogenesis [24,25]. In order to test whether sRNA23 is involved in the response to different microenvironmental signals, different concentrations of BIP, NaCl, paraquat, and lysozyme were added to the normal medium *in vitro* to create an environment under nutrient, hypertonic, oxidative, and lysozyme stress. The expression of sRNA23 was monitored by Northern blot. We found that the expression of sRNA23 gradually decreased with the increase in BIP concentration (Figure 4(a)), which was consistent with the results of RT-PCR and RNA-seq. Similarly, in paraquat induced oxidative stress, the expression of sRNA23 decreased in a dose-dependent manner (Figure 4(b)). Conversely, the expression of sRNA23 was up-regulated in a dose-dependent manner under hypertonic (NaCl) and lysozyme stress conditions (Figure 4(c,d)). Our data indicate that *S. suis* express sRNA23 differentially against different stress conditions, which may be beneficial to the survival of *S. suis* under different stress environments.

### Deletion of sRNA23 has a profound effect on cell morphology and biofilm formation

**Figure 5.**
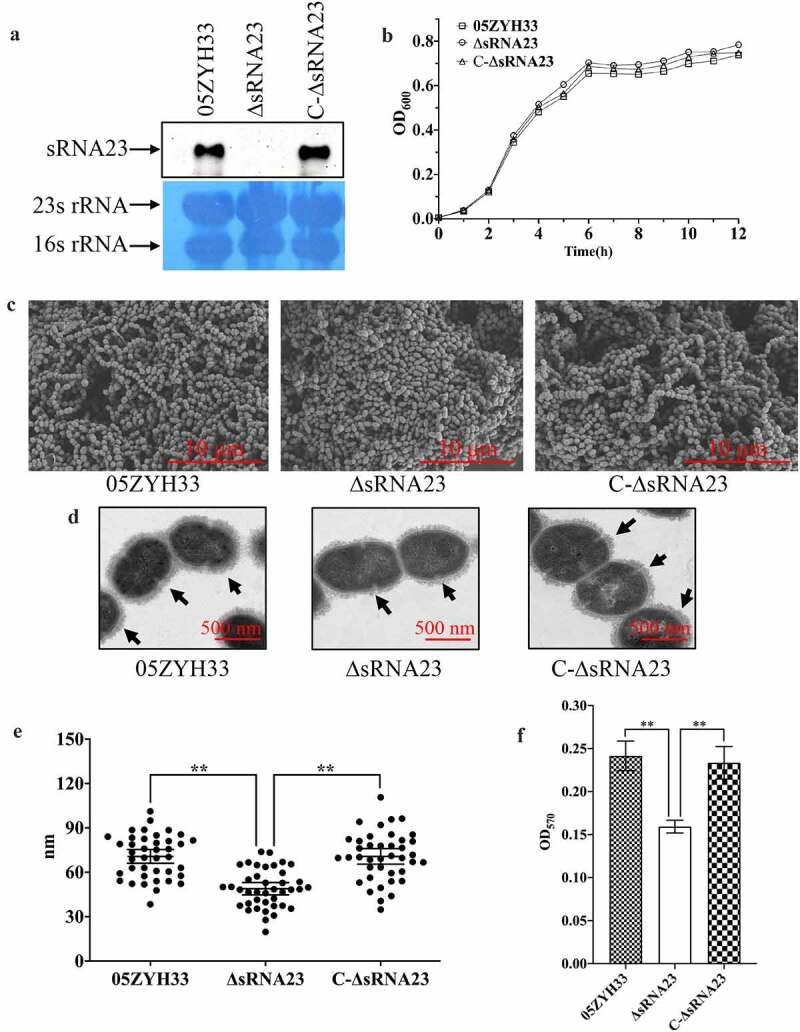
Deletion of sRNA23 has profound effects on cell morphology and biofilm formation. (a) Northern blot analysis for detection of sRNA 23 in 05ZYH33, sRNA 23 deletion strain (ΔsRNA23), and complementation strain (C-∆sRNA23). (b) The OD_600_ of 05ZYH33, ΔsRNA23, and C-∆sRNA23 cultured in THB + 5% FBS were detected at different time (n = 3). (c) Cell morphology was examined by scanning electron microscope (scale bar, 10 μm). (d) Transmission electron microscope was performed to compare the CPS thickness of 05ZYH33, ΔsRNA23, and C-∆sRNA23. The scale bar indicates 500 nm. (e) The capsular thickness of each strain was quantified using Image J 1.50 software. Data are shown as means ± SD (**P< 0.01). (f) Reduction of biofilm formation by sRNA23 inactivation in 05ZYH33. The spectrophotometer UV-5500 (Shanghai Metash Instruments) was applied to measure optical density at 570 nm (OD_570_) here. The data are presented as means ± SD (**P< 0.01)

In order to explore the potential effects on cell morphology and biofilm-forming ability of sRNA23, *S. suis* isogenic sRNA23 deletion and complementing C-ΔsRNA23 strains were constructed, and the constructs were tested by Northern blot (Figure 5(a)). The effects of sRNA23 deletion on morphological characteristics of *S. suis* were observed by Gram staining, scanning electron microscopy (SEM), and transmission electron microscopy (TEM). We observed that there was no significant difference in the growth rate among ΔsRNA23, WT, and C-ΔsRNA23 (Figure 5(b)). Gram staining results showed that the inactivation of sRNA23 resulted in shortening of the bacterial streptococcal chain length and as expected, complementation restored the phenotype of *S. suis* (Figure S2). Next, SEM demonstrated that sRNA23-deleted mutants lost the ability to form streptococcal chains (Figure 5(c)). Bacterial chain length is also an important virulence factor that enhances adherence and colonization to the host [26,27]. TEM of capsular polysaccharide showed that the thickness of the capsular polysaccharide of ΔsRNA23 is significantly thinner than that of WT and C-ΔsRNA23 (*P* < 0.01) (Figure 5(d,e)). Finally, biofilm formation was monitored using crystal violet staining and quantified. As shown in Figure 5(e), the ΔsRNA23 strain exhibited a significantly weakened ability of biofilm formation compared with WT and C-ΔsRNA23 strains. These results indicate that the lack of sRNA23 leads to shorter chain length, thinner capsular polysaccharide, and weaker biofilm formation ability.

### sRNA23 deletion impairs adhesion & hemolytic activity and enhances susceptibility toward phagocytosis

**Figure 6.**
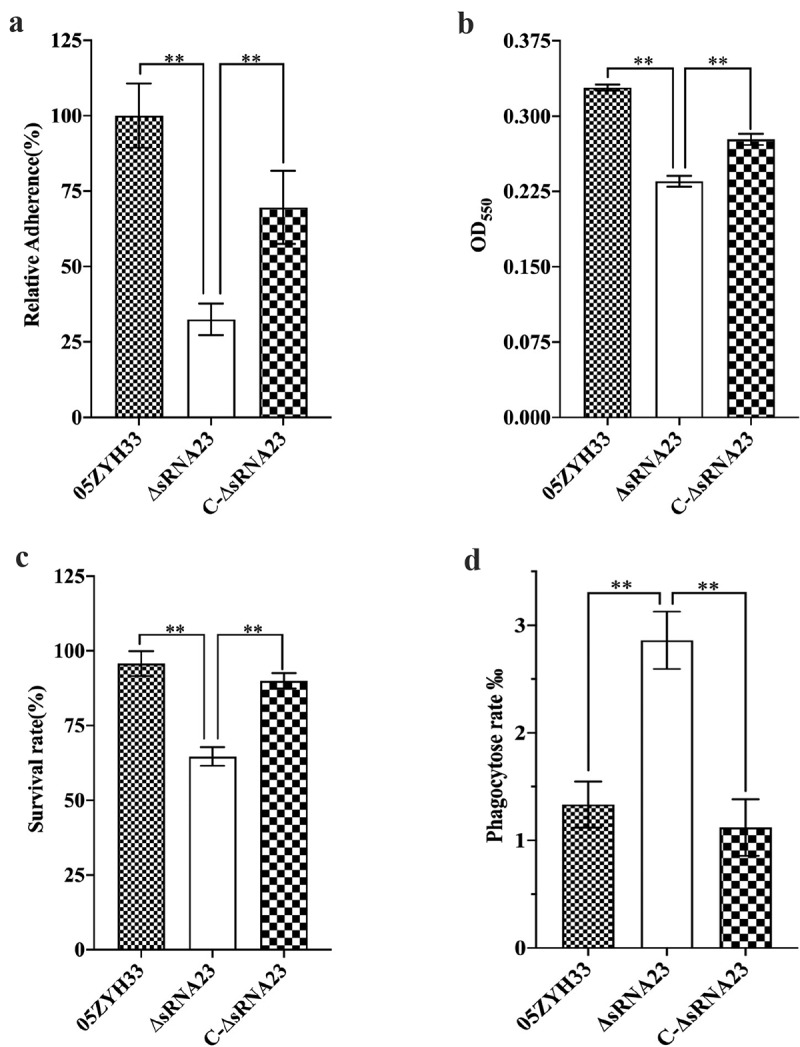
sRNA23 deletion impairs adhesion & hemolytic activity, and enhances susceptibility toward phagocytosis. (a) Relative adherence of the WT strain 05ZYH33, ∆sRNA23 mutants and complementation strain C-∆sRNA23 on HEp-2 cells was analyzed by bacterial adhesion assay (**P< 0.01). (b) Hemolytic activity analysis of *S. suis* WT strain (05ZYH33), ∆sRNA23, and C-∆sRNA23(**P< 0.01). (c) Survival rate of indicated *S suis* strains in pig blood (**P< 0.01). (d) Phagocytosis rate of indicated *S suis* in RAW264.7(**P< 0.01)

Effective adhesion to the surface of host cells is a prerequisite for bacteria to invade, colonize, multiply, and spread [28]. HEp-2 cells were used to evaluate whether sRNA23 affects the adhesion ability of *S. suis*. Compared with WT, the adhesion ability of ΔsRNA23 strain to HEp-2 cells was significantly reduced by 70% (*P* < 0.01) (Figure 6(a)). At the same time, we determined the erythrocyte hemolytic activity of the supernatants of *S. suis* 2 strains. As shown in Figure 6(b), the hemolytic activity of ΔsRNA23 was significantly lower than WT, indicating that sRNA23 is involved in the regulation of hemolytic activity in *S. suis. S. suis* often causes sepsis, and the ability to survive and grow in whole blood is important for *S. suis* to cause infection [29]. We examined the ability of 05ZYH33, ΔsRNA23, and C-ΔsRNA23 strains to survive in phagocytic cells such as pig whole blood and RAW264.7 cells. The survival rate of the WT strains in pig whole blood was significantly higher than ΔsRNA23 strains (Figure 6(c)). Once *S. suis* spreads into deep tissues or blood, phagocytosis plays an important role in the host’s defense against invading pathogens [30]. We utilized RAW264.7 to study the role of sRNA23 in *S. suis*-phagocyte interaction. As shown in Figure 6(d), ΔsRNA23 was more likely to be phagocytosed by RAW264.7 cells than 05ZYH33. These results indicate that sRNA23 inactivation renders *S. suis* more susceptible to phagocytosis.

### sRNA23 interacts with FBA and rplB

**Figure 7.**
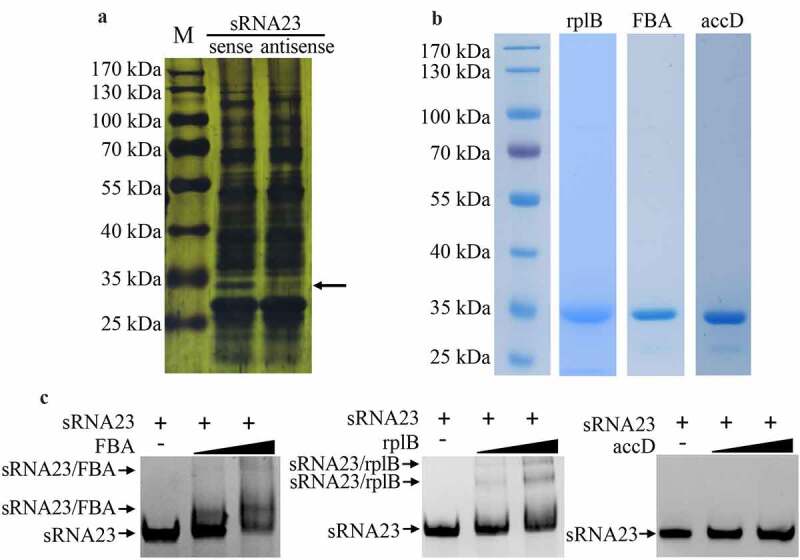
sRNA23 interacts with FBA and rplB. (a) Silver staining of proteins pulled down by sRNA23 sense probes and sRNA23 antisense probes from *S. suis* 05ZYH33 total protein. The special sRNA23 associated band (arrow) was excised for mass spectrometry. (b) Indicated candidate proteins were expressed in *E. coli* 21(DE3) and purified by Ni-NTA resin. (c) The binding ability of these purified proteins to sRNA23 was determined by EMSA (FBA 0, 1 μg, 2 μg; rplB 0, 0.5 μg, 1 μg; accD 0, 1 μg, and 2 μg)

Knowing that sRNAs can regulate bacterial virulence by targeting mRNAs or directly binding with proteins, we conducted bioinformatics screen to predict the target mRNAs of sRNA23 by two bioinformatic tools (Table S3 and Figure S3). We selected 26 genes as candidate targets of sRNA23 and subjected for RT-PCR to examine relative transcription of predicted target mRNA genes in WT and ΔRNA23 mutant. However, of 26 predicted genes, only one gene showed a noticeable decreased expression in ΔRNA23 compared to WT (Figure S3). Next, an RNA pull-down experiment was performed using biotinylated sRNA23 as a bait to explore the proteins that potentially interact with sRNA23. Taking the reverse complementary strand of sRNA23 as a control, the product of the RNA pull-down was separated by SDS-PAGE and then silver stained. Silver staining showed a specific separated protein band (Figure 7(a)). The specific band was cut and analyzed by mass spectrometry. Based on mass spectrometry and computational genome annotation, we identified that three proteins could potentially interact with the sense strand sRNA23 (Table S4). Therefore, these three proteins were expressed in *E. coli* by recombinant DNA technology, and recombinant proteins were purified by nickel affinity chromatography (Figure 7(b)). The binding ability of these three proteins with the sense strand of sRNA23 was verified by electrophoretic mobility shift assay (EMSA) (Figure 7(c)). EMSA results reveal that sRNA23 can interact with FBA (Fructose bisphosphate aldolase) and rplB (50S ribosomal protein L2).

## Discussion

*S. suis* is an important zoonotic pathogen responsible to cause huge economic losses to the global pig industry and can also seriously harm human health. The pathogenic process of *S. suis* includes colonization, invasion, and followed by systemic infection. During infection, *S. suis* can inevitably be exposed to various stress environments, such as iron and nutritional starvation, oxidative and acidic stress, and so forth [31,32]. Iron is an essential metal element for the survival, multiplication, and pathogenesis of *S. suis* [33]. Over the past decade, sRNAs have been identified in a wide range of bacteria and found to play critical regulatory roles in bacterial life processes including bacterial pathogenesis [34,35]. Iron uptake by bacteria is essential for pathogenesis. Several sRNAs have been reported to regulate iron uptake to enhance bacterial pathogenicity [36–40]. In this study, an *in vitro* medium was treated with an iron chelator (BIP) to create an iron starvation environment. Iron starvation caused *S. suis* to induce differential expression of several sRNAs. Of these differentially expressed sRNAs, we identified and characterized a novel sRNA, referred to as sRNA23 implicated in the pathogenesis of *S. suis*. We found that deletion of sRNA23 in *S. suis* resulted in decreased adherence to the HEp-2 cells, increased sensitivity to phagocytosis by RAW264.7, and significantly reduced hemolytic activity. Furthermore, the sRNA23-deleted mutant had low survival rate in pig whole blood and attenuated virulence in a mouse model. These results indicate that sRNA23 plays an important role in enhancing the pathogenesis of *S. suis* 2.

Recently, several sRNAs of *S. suis* 2 have been identified through RNA-seq or comparative genome analysis as potential regulators that might be involved in bacterial pathogenesis. However, the precise mechanisms of how these sRNAs are implicated in bacterial pathogenesis are largely unknown [16–18]. In this study, 14 sRNAs were identified as potential regulators by RNA-seq. Of them, 10 sRNAs were previously uncharacterized in the studies related to *S. sui*s [18]. The expressions of sRNAs were also validated by RT-PCR. Identification and characterization of new regulatory sRNAs can help understand how pathogens respond to various host’s microenvironments for their survival and the causation of disease. The expression of sRNAs is often altered by multiple host-like stressors in vitro [41]. Northern blot detected the abundance and expression of sRNA23 under different stress conditions. Expression of sRNA23 was downregulated under iron deprivation medium, which was consistent with the results of RNA-seq. In addition, like many sRNAs, sRNA23 expression was induced by oxidative and lysozyme stress [42,43]. These findings suggest that differential expression of sRNA23 to different environments may be beneficial for bacterial adaptation and pathogenesis. In a mouse model, the ΔsRNA23 strain was less virulent than the WT strain, indicating that sRNA23 can play an important role in the pathogenesis of *S. suis* 2. The pathogenic process of *S. suis* includes adhesion, colonization, invasion, proliferation, and toxin secretion. The virulence factors secreted by *S. sui*s in the process during invasion and proliferation would damage host cells or tissues, or induce host cells to produce inflammatory factors to change the permeability of epithelial cells, eventually leading to sepsis and meningitis [44]. In this study, the ΔsRNA23 strain showed a significant reduction in the ability to adhere to HEp-2 cells, and increased sensitivity to RAW264.7 macrophages. Others sRNAs are reported to modulate pathogenic process by multiple mechanisms such as enhancing adherence to host cells, increasing resistance to phagocytosis, involving in biofilm formation and so forth [12,16,18,45,46]. Herein, ΔsRNA23 strain also demonstrated a reduced biofilm formation ability and the capsular polysaccharide formed by ΔsRNA23 strain was significantly thinner than WT strain. These results indicate that sRNA23 plays an important role in the process of *S. suis* pathogenesis at least by enhancing adherence to host and resistance to phagocytosis.

To uncover the in-depth mechanism how sRNA23 is implicated in S suis2 pathogenesis, we conducted bioinformatics screen to predict target mRNAs of sRNA23 by two bioinformatic tools. We selected 26 genes as candidate targets of sRNA23 and subjected for RT-PCR to examine relative transcription of predicted target mRNA genes with (WT) or without (ΔRNA23) sRNA23. Of them, only one gene i.e., *tsaD* (Gene ID: SSU05_0166) showed a noticeable decreased expression in ΔRNA23 compared to WT through RT-PCR analysis. In bacteria, tsaD is one of the important proteins involved in the catalysis of N(6)-threonylcarbamoyladenosine (t6A) biosynthesis [47]. Universally conserved t6A modification of tRNA is central to translational fidelity [48]. However, the exact role of *tsaD* in bacterial pathogenesis is yet to explore. Future study will explore biological consequences resulted from sRNA23-*tsaD* regulatory network including bacterial pathogenesis.

Because our RT-PCR results revealed that sRNA23 might not have a profound effect on the expression of the vast majority of predicted mRNAs, we speculated sRNA23 would have regulatory role by binding proteins. sRNA possesses intrinsic dynamic structures which are used by proteins to promote specific interactions and trigger biological responses. Proteins that can bind sRNA include RNA chaperones, enzymes, and RNA scaffolds [49]. In most bacteria, typical RNA binding proteins such as Hfq or CsrA have well-defined RNA-binding domains and sRNAs usually bind with these proteins to drive the regulatory activity [50]. Recently, it was reported that some RNA binding proteins, unlike typical RNA binding proteins, do not have well-defined RNA-binding domains; however, they play an important role in adaptation to harmful environments [51]. For example, the SpoVG protein in Gram-positive bacteria could interact with at least three sRNAs and participate in response to lysozyme resistance, swarming motility, and virulence [52]. RNA affinity chromatography, RNA co-immunoprecipitation, and RNA pull-down are usually used to identify protein binding partners. In *Staphylococcus aureus*, 7 proteins that bind to RNAIII were identified by pull-down technology using RNAIII as bait [53]. In the present study, to reveal the possible molecular mechanism of sRNA23, we used RNA pull-down, mass spectrometry and EMSA to investigate the proteins that bind sRNA23. We found that FBA and rplB could associate with sRNA23. These results indicate that sRNA23 may involve in the pathogenesis of *S. suis* by binding to the target protein.

FBA, which we found as a sRNA23 interactor, is not only a core enzyme involved in the glycolysis and gluconeogenesis pathway, but also a key surface protein in bacteria which has been reported to play an important role in the pathogenesis of several pathogens. For example, FBA enhances adherence to the host and regulates the fibronectin-mediated immune response [54,55]. FBA appears to be essential for bacterial survival because it cannot be successfully deleted in many bacteria [56,57]. In *Streptococcus pneumoniae* and *Mycoplasma hyopneumoniae*, anti-FBA antibodies can inhibit the adhesion and pathogenicity to epithelial cells [57,58]. The FBA of *Neisseria meningitidis* is located in the cytoplasm and outer membrane, and binds to human cells by associating with plasminogen [59]. In the chronic stage of *M. tuberculosis* infected mice, FBA is required for survival [60]. It has also been demonstrated that FBA is essential for the replication and virulence of *Toxoplasma gondii* [61]. In addition, FBA is required for the efficient multiplication of *Francisella* in macrophages and it regulate pathogenesis [62]. *Klebsiella pneumoniae* exhibited a significant reduction in phagocytosis and lethality of neutrophils after treatment with FBA inhibitors or FBA-antibodies [63]. FBA is highly conserved in *S. suis*. In *S. suis* 9, an immunoproteomic analysis showed that FBA is located on the cell surface, possesses immunogenic activity, and may be involved in adaptation to stress environment [64]. rplB is one of the proteins on the 50S large subunit of the ribosome, located at the interface of the two subunits of the size 50S and 30S. It is located at the most important part of the active region of peptide acyltransferase and is an indispensable protein for protein translation [65]. Bacteria have undergone significant changes from planktonic growth to biofilm formation, and many genes can change expression during this process. The rplB of *Escherichia coli* and *Helicobacter pylor*i have significantly downregulated during biofilm formation [66,67]. Therefore, it can be speculated that sRNA23 may enhance the adhesion ability to the host and anti-phagocytosis by binding to FBA and possibly regulate the formation of biofilm by binding to rplB, thereby affecting *S. suis* 2 pathogenesis. However, the specific mechanism of how sRNA23 and FBA/rplB binding regulates *S. suis* pathogenesis remains to be elucidated. Further studies are necessary to explore the consequences of this binding in *S. suis* pathogenesis.

In conclusion, we identified 10 novel sRNAs through prokaryotic strand-specific transcriptome sequencing in *S. suis* 05ZYH33. We found that the newly identified sRNA23 plays a critical role in regulating pathogenic processes such as adhesion, anti-phagocytosis and biofilm formation in *S. suis* 2. Furthermore, we found that sRNA23 binds to FBA and rplB and this binding might play important role in the pathogenesis of *S. suis* 2. The study of the regulatory mechanism of sRNA23 in the pathogenesis of *S. suis* will help to explore the virulence regulatory network of *S. suis* and provide a novel theoretical basis for the prevention and control of *S. suis* infection.

## Materials and Methods

### Bacterial strains and culture conditions

*S. suis* strains were maintained and grown in Todd-Hewitt broth (THB; OXOID, England) plus 5% fetal bovine serum (THB + 5% FBS) at 37°C. Solid media contained 2% agar. *S. suis* 05ZYH33 (isolated from a clinical patient with meningitis) and *S. suis* SC19 (isolated from a clinical diseased pig) were kindly provided by Professor Y. Feng (Zhejiang University) and Professor C. Tan (Huazhong Agricultural University), respectively. *Escherichia coli* strains Top10 and BL21(DE3) were cultured in Lysogeny Broth (LB) medium or plated on LB agar at 37°C. When required, differential final concentrations of antibiotics (Sangon, Shanghai, China) were added into the medium as follows: for *S. suis*, spectinomycin at 100 μg/mL; for *E. coli*, spectinomycin at 50 μg/mL, ampicillin at 100 μg/mL, and kanamycin at 50 μg/mL.

### Strand-specific dUTP library preparation for Illumina sequencing

For deep sequencing, an overnight culture of the 05ZYH33 strain was diluted 1:100 in fresh medium to achieve an initial OD_600_ of 0.04. After 30 min of culture with shaking, the cultures were supplemented with 2 mM BIP or ethanol. After 60 min of treatment, bacterial cells were collected by centrifugation at 12,000 × g for 5 min. The cell pellets were immediately used for RNA extraction. cDNA library preparation and sequencing were performed with the assistance of Majorbio Biopharm technology Co., Ltd (Shanghai, China).

### Construction of deletion and complementation strains

Deletion of sRNA23, sRNA24, and sRNA76 in 05ZYH33 were carried out by homologous recombination using the thermosensitive suicide *S. suis-E. coli* vector pSET4s [68]. Briefly, the left-arm and right-arm flanking regions (LA and RA) of the target gene (~1,000 bp) were amplified using specific primers enlisted in Table S5. The resulting left-arm fragment and right-arm fragments were inserted into a linearized vector (*BamH* I and *EcoR* I) using the ClonExpress II one-step cloning kit (Vazyme, China). The recombinant vectors were transformed into competent cells 05ZYH33 by electroporation. After two steps of allelic exchange, the putative mutant strains were screened by PCR using primers listed in Table S5 and confirmed by DNA sequencing. The mutants were also further confirmed by Northern blot. The recombinant pSET2-sRNA23 plasmid containing the full DNA fragment of sRNA23 and the upstream 200 nt was electroporated into ΔsRNA23 competent cells.

### Morphological observation of the 05ZYH33 and mutant strain

The strains were inoculated into THB + 5% FBS, and cultured to mid-exponential phase (OD_600_ ≈ 0.6) at 37°C, and then washed with sterile water. Each sample was dropped on glass slides and fixed by flaming. Gram staining was performed according to the manufacturer’s instructions provided in the Gram staining kit (Solaibo, China). The morphology of bacteria was observed and photographed under a light microscope (10 × 100 times). Scanning electron microscopy (SEM) and transmission electron microscope (TEM) assays were performed by previously described methods [69]. 05ZYH33, ΔsRNA23, and C-ΔsRNA23 were harvested at mid-exponential phase (OD_600_ ≈ 0.6) and fixed with 2.5% glutaraldehyde at 4°C overnight. After washing twice with PBS, they were postfixed with 1% osmium tetroxide in PBS for 1–2 h at room temperature. The subsequent dehydration was conducted by a graded series of ethanol (30%, 50%, 70%, 80%,90%, 95%, and 100%) for 15 min at each step. The samples were subjected to dehydration in critical point dryer and sputter-coated with gold for 30 s, and finally scanned using Hitachi SU-8100 SEM (Hitachi, Japan). For the TEM study, ethanol dehydrated samples were transferred into absolute acetone for 20 min at RT, embedded in Spurr resin and sectioned using Leica UC7 ultramicrotome. The ultrathin sections were stained with 2% uranyl acetate (8 min) and 2.6% alkaline lead citrate (8 min), and observed in Hitachi HT-7800 TEM (Hitachi, Japan). Statistical analysis was performed by ImageJ.

### Survival in swine whole blood

Bacterial survival rate in whole blood was performed by previously described methods [69]. 05ZYH33, ΔsRNA23, and C-ΔsRNA23 were cultured in THB + 5% FBS to the mid-exponential phase (OD_600_ ≈ 0.6). Bacteria were collected and adjusted to 0.01 at OD_600_ with PBS. Subsequently, 900 μL of fresh blood were mixed with 100 μL bacterial suspension and incubated for 3 h at 37°C with gentle shaking. At 0 h and 3 h incubation, the mixtures were serially diluted, vortexed, and plated onto THB + 5% FBS plates to determine the survival bacteria. Survival rates were calculated as follows: (recovered CFU at 3 h)/(CFU at 0 h)×100%. Three independent experiments were performed in duplicates.

### Hemolytic activity detection

Hemolytic activity (HA) was detected in accordance with previously described methods [70]. Briefly, the supernatant was collected from the mid-exponential phase (OD_600_ ≈ 0.6) by centrifugation at 12,000 × g for 5 min. Each 50 μL of the supernatant was incubated with 150 μL 2% swine red blood cells (prewashed twice with PBS) for 2 h at 37°C. Unlysed erythrocytes were removed by centrifugation at 1,000 × g for 10 min. The supernatants (150 μL each) were transferred to a new 96-well microplate to measure the OD_550_ value. Three independent experiments were performed in duplicates.

### RNA extraction and RT-PCR

Total RNA was extracted for RT-PCR, RACE, and Northern blot as follows: Bacteria were grown in THB + 5% FBS at 37°C to mid-exponential phase or appropriate time (OD_600_ = 0.3, 0.6, 0.8) and collected by centrifugation. Cells were treated with lysozyme at 37°C for 15 min, then total RNA was extracted using TRIzol according to the instructions. Genomic DNA was removed by using RNase-free DNase I (Takara, Japan) at 37°C for 3 h. Treated RNA was purified by phenol-chloroform extraction and ethanol precipitation, dissolved in DEPC-treated water, and stored at −80°C until use. The RNA quality was examined by gel electrophoresis and RNA concentrations were determined using a nanodrop spectrophotometer (Eppendorf, Germany). The primers used for RT-PCR with cDNA templates are listed in Table S5. The amplification RT-PCR products were separated by 2% agarose gel electrophoresis.

### Biofilm assay

Biofilm formation by the three strains was performed according to previously described methods with some modifications [71]. Briefly, bacteria were grown in THB + 5% FBS at 37°C to mid-exponential phase and then were diluted 1:100 in either fresh THB + 5% FBS. The diluted cultures (200 μL) were added to a 96-well microplate. After incubation at 37°C for 24 h, the culture medium and unattached bacteria were removed and washed three times with sterile water. After air drying, 1% crystal violet (200 μL) was added to stain the biofilms for 30 min. After washing three times with sterile water, and complete drying, 95% ethanol (200 μL) was added to each well to dissolve the crystal violet and incubated for 30 min. Finally, 100 μL from each well were transferred to a new 96-well microplate and quantified by recording the absorbance at 570 nm. The experiments were done in triplicate and repeated at least three times.

### Lysozyme, BIP, osmotic, and oxidative stress assays

To evaluate the sensitivity of *S. suis* to lysozyme, bipyridine, osmotic (NaCl) and oxidative (paraquat) stress, mid-exponential phase (OD_600_ ≈ 0.6) cultures were treated with serial dilutions of lysozyme (0, 0.25, 0.5, and 1 mg/mL), bipyridine (0,1, 2, 4 mM), NaCl (0,1, 2, 4 mM) and paraquat (0, 2.5, 5.0, 10 mM) for 1 h, then, samples were collected and subjected to total RNA extraction. The sRNA23 expression level was detected by Northern blot. The experiments were repeated three times.

### Adhesion to epithelial cells and anti-phagocytosis assay

Bacterial adhesion assay was performed on HEp-2 cells according to the previously described procedure [70]. The anti-phagocytosis ability was assessed using RAW246.7 cells, as described previously [70]. Bacteria were harvested by centrifugation at mid-exponential growth phase (OD_600_ ≈ 0.6), washed twice with PBS, and resuspended with DMEM culture medium without antibiotics. Then the bacteria were poured into 24-well cell plates containing HEp-2 cells at a rate of 50:1. The plates were centrifuged at 800 × g for 10 min to bring the bacteria into contact with the cells. After incubation at 37°C for 2 h, the infected cells were washed three times with PBS and resuspended with 500 μL of lysis buffer. Additionally, for anti-phagocytosis, the cells were treated with penicillin (5 μg/mL) and gentamycin (100 μg/mL) (Solaibo, China) for 1 h to eliminate extracellular bacteria. The numbers of adherent or phagocytic bacteria were enumerated by plating 10-fold serial dilutions on the THB agar plates. Each assay was performed thrice independently.

### Virulence assay

To compare the pathogenicity of the ΔsRNA and 05ZYH33 strains a survival rate assay was carried out as described in previous studies [18,72]. Five-weeks-old specific pathogen-free female BALB/c mice were randomly divided into four groups (10 mice per group) and infected with WT or mutant strains (05ZYH33, ΔsRNA23, ΔsRNA24, and ΔsRNA76) at a dose of 5 × 10^8^ CFU via intraperitoneal injection. The negative control group was injected with an equal volume of sterile PBS. Survival rates were measured for 7 days and repeated three times independently.

### 5′RACE and 3′RACE

For generation of the full-length of sRNA23, 5′and 3′RACEs were performed using the SMARTer 5′/3′ RACE Kit (Clontech, Japan) following the manufacturer’s instructions. The gene-specific primers for 5′ and 3′RACEs are listed in Table S5. Before initiating the 3′RACE, total RNA was polyadenylated with *E. coli* poly (A) polymerase (NEB, USA) at 37°C for 10 min. The PCR products were gel purified, cloned into the pEASY-Blunt vector (TransGen, Beijing, China), and sequenced with M13F primer. Eight clones were randomly chosen and sequenced individually for each RACE, and the farthest sequence of the 5′ or 3′ end was considered the 5′ or 3′ end of the sRNA.

### In vitro RNA transcription

The template for sRNA23 transcription was amplified by PCR with T7 promoters. The single-strand RNAs were produced by T7 high-efficiency transcription kit (TransGen, Beijing, China) and then were purified by magnetic beads.

### Northern blot analysis

A dig-labeled sRNA23 DNA probe was generated using the DIG High Prime labeling kit (Roche, Switzerland). Northern blot was done by using the DIG Northern Starter Kit (Roche, Switzerland) according to the manufacturer’s protocol. Briefly, 20–25 μg of total RNA were denatured at 65°C for 10 min, and immediately placed on ice for 5 min, followed by separation on formaldehyde denaturing 1.2% agarose gel. RNA samples were then transferred to Amersham Hybond-N^+^ membrane (GE Healthcare, USA) via capillary in 20× SSC, the membrane was cross-linked under UV light, and ribosomal RNA bands were visualized using methylene blue staining. After a prehybridization for 60 min at 65°C, DIG-labeled DNA probes were added to the hybridization buffer to perform hybridization overnight at 65°C. After washing and blocking, the membrane was incubated with anti-digoxigenin-AP (1:10^4^ in blocking buffer) for 30 min at room temperature. The membrane was washed, equilibrated in 1× Detection buffer for 5 min, covered with CDP-Star solution for 5 min, and signals developed were recorded by the chemiluminescence imaging system.

### RNA pull-down assay

RBPs pull-down assay was performed following the user guidelines of Magnetic RNA-Protein Pull‐Down Kit (Thermo Scientific Pierce, Waltham, USA), with some modifications. Briefly, *S. suis* cultures (20 mL) were collected and bacterial cells were resuspended with 1.5 mL phosphate buffer (50 mM, PH7.0), and sonicated for 15 min. After centrifugation at 8,000 × g for 10 min, the supernatants were collected and the concentration of total protein was quantified via BCA assay. About 100 μg protein per sample was used for pull-down assay. At the same time, 5 μg of bait RNA sample were denatured at 85°C for 5 min and then cooled immediately on ice. RNAs were labeled by desthiobiotinylation with the Pierce RNA 3′End Desthiobiotinylation Kit (Thermo Scientific Pierce, Waltham, USA), then incubated with Streptavidin Magnetic Beads. Then, these beads were mixed with total protein in RIP buffer at 4°C with agitation or rotation for 1 h to achieve RNA-binding proteins. After washing with RIP wash buffer three times, the final retrieved proteins were boiled in SDS loading buffer, separated by SDS-PAGE, subjected to silver staining, and the band of interest was excised and subjected to in-gel trypsinization. The tryptic peptides were analyzed by LC-MS/MS. Peptide mass fingerprint and sequence data were analyzed using the UniProt and NCBI databases.

### Expression and purification of recombinant proteins

*fba, accD* and *rplB* were amplified from *S. suis* SC19 genomic DNA by PCR with the primers listed in Table S5. The PCR products were inserted into linearized pET28a vector (digested with *Nco* I and *EcoR* I) by homologous recombination, then transformed into *E. coli* Top 10. For protein expression, pET28a-*fba*, pET28a-*accD*, and pET28a-*rplB* were transformed into *E. coli* BL21(DE3), then incubated in LB broth supplemented with 50 μg/mL kanamycin and 0.05 mM IPTG at 16°C for 15 h. The recombinant proteins were purified by Ni-NTA resin (Sangon, Shanghai, China), and imidazole was removed through ultrafiltration. SDS-PAGE and the BCA assay were used to determine protein purity and concentration.

### RNA electrophoretic mobility shift assay

The RNA was denatured at 80°C for 10 min and then quickly cooled on ice. A total of 500 ng sRNA23 were incubated with increasing amounts of proteins in RNA EMSA Binding Buffer (10 mM HEPES, pH = 7.3, 20 mM KCl, 1 mM MgCl_2_,1 mM DTT, 5% glycerol, 50 ng/mL BSA) in 10 μL mixture at room temperature for 30 min. The RNA-protein complexes were then separated by electrophoresis in 4% nondenaturing polyacrylamide gels in 0.5 × TBE for 90 min. The gels were stained with 0.5 μg/mL ethidium bromide dye at room temperature for 30 min and washed in 50 mL DEPC-treated water at room temperature for 10 min. The images were captured using a Bio-Rad gel imaging system.

### RNA secondary structure prediction

The RNAfold program (http://rna.tbi.univie.ac.at/) was used to predict the most stable RNA secondary structure based on the lowest folding energy.

### Statistical analysis

Statistical analysis was performed using a two-tailed unpaired Student’s t-test in GraphPad prism 7. All of the data were expressed as mean ± standard deviation (SD). Differences were considered statistically significant at a value of *P* < 0.05.

## Supplementary Material

Supplemental Material

## Data Availability

The datasets produced in this study are not available publicly because they are currently private (SRA accession number: PRJNA717999) and are scheduled to be released on 30 December 2021. Any requests to access the datasets should be directed to YC.
