# Supplementary material for: sRNA23, a novel small RNA, regulates to the pathogenesis of Streptococcus suis serotype 2
Source: Virulence. 2021 Dec 9;12(1):3045–61. doi: 10.1080/21505594.2021.2008177 (PMC8667912; doi:10.1080/21505594.2021.2008177)
Supplement: Supplemental Material [file KVIR_A_2008177_SM9937.zip › supplementary/Supplementary materials.docx]

**Supplementary materials**

**Figure S1.** Schematic diagram showing construction map of ΔsRNA strains by allelic replacement. Primers for Left Arm (LA)-F/R, Right Arm (RA)-F/R, M13F/In-R, and Out-F/R used for the construction and detection of sRNA mutants are listed in Table S5.

**Figure S2.** Gram-stained images of *S. suis* 2 strains under light microscopy.

**Figure S3.** RT-PCR verification of predicted target mRNAs of sRNA23. RNA was isolated from 05ZYH33, ΔsRNA23 and C-ΔsRNA23 and relative transcription of predicted target mRNA genes was quantified and normalized with 16s rRNA
