# Supplementary material for: sRNA23, a novel small RNA, regulates to the pathogenesis of Streptococcus suis serotype 2
Source: Virulence. 2021 Dec 9;12(1):3045–61. doi: 10.1080/21505594.2021.2008177 (PMC8667912; doi:10.1080/21505594.2021.2008177)
Supplement: Supplemental Material [file KVIR_A_2008177_SM9937.zip › supplementary/Table Supplement_clean.docx]

| **Table S1. The predicted sRNAs in *S.suis* 05ZYH33 by RNA-seq** | | | | |
| --- | --- | --- | --- | --- |
|  | Start | End | Strand | Antisense_Genes |
| sRNA11 | 200763 | 200886 | - | - |
| sRNA14 | 356424 | 356688 | + | - |
| sRNA16 | 414200 | 414329 | + | - |
| sRNA19 | 501007 | 501124 | + | - |
| sRNA2 | 26407 | 26509 | + | - |
| sRNA23 | 618465 | 618969 | + | - |
| sRNA24 | 619214 | 619669 | + | - |
| sRNA28 | 683924 | 684250 | + | - |
| sRNA3 | 58446 | 58734 | + | - |
| sRNA31 | 730075 | 730264 | + | - |
| sRNA40 | 1122816 | 1122980 | - | - |
| sRNA54 | 1513729 | 1514002 | + | - |
| sRNA71 | 1851057 | 1851169 | - | - |
| sRNA76 | 1989584 | 1989895 | - | - |

**Table S2. Full-length sequence of** **sRNA23, Related to Figure 3A**

| sRNA23 |
| --- |
| ACGTTTCCGATAGGAGGAAGCAAATATCTATACAAAGGAGATGTTTCATCATGAAACATATTGTAACAAAACGTAATTATAAAGTTGGCGCATGCGCTATGGCACTTGCATTGTTGATGACTACTGCAGGTGGTGCAGTTGTAAATGCAGAGCAGCACCTTGAGTCATCAGTTGGCTTAGTACAGCGTGGTGATGTACTTCTAAAATTGAAAGAATCAAACCAGTCTTTCAATGAAGGAGAGGTTGGAAATTTAGAGCCTGTCAACCCAACTGTGCCTCCAATGGCTTCTTCTGTACAAAAA |

The full-length sequence of sRNA23 is combined the end sequences of sRNA23 determined by 5′ and 3′ RACE

**Table S3. Prediction of sRNA23 target genes by TargetRNA2 and RNAPredator**

| Gene_ID | Description | TargetRNA2 | | | | | |  | RNAPredator | | | | | |
| --- | --- | --- | --- | --- | --- | --- | --- | --- | --- | --- | --- | --- | --- | --- |
|  |  | Energy | Pvalue | sRNA_start | sRNA_stop | mRNA_start | mRNA_stop |  | Energy | Z-score | sRNA_start | sRNA_stop | mRNA_start | mRNA_stop |
| SSU05_1689 | Dpr | -14.2 | 0.001 | 41 | 54 | -2 | 12 |  | -8.21 | 0.25 | 89 | 115 | 327 | 354 |
| SSU05_1473 | SAM-dependent methyltransferase | -13.5 | 0.003 | 127 | 139 | -32 | -20 |  | -3.95 | 2.01 | 124 | 138 | -30 | -17 |
| SSU05_1922 | endo-beta-N-acetylglucosaminidase, putative | -13.3 | 0.003 | 82 | 96 | -75 | -61 |  | -14.5 | -2.36 | 268 | 292 | 2404 | 2427 |
| SSU05_0048 | ABC-type multidrug transport system, ATPase and permease component | -13.3 | 0.003 | 12 | 24 | -1 | 12 |  | -9.39 | -0.24 | 255 | 263 | 902 | 910 |
| SSU05_2181 | Uncharacterized conserved protein | -12.9 | 0.004 | 8 | 22 | -73 | -59 |  | -8.97 | -0.07 | 283 | 297 | 214 | 228 |
| SSU05_0166 | Metal-dependent proteases with possible chaperone activity | -12.7 | 0.005 | 40 | 54 | -2 | 13 |  | -15.5 | -2.78 | 275 | 298 | 183 | 208 |
| SSU05_0024 | Fatty acid/phospholipid biosynthesis enzyme | -12.6 | 0.005 | 120 | 130 | 10 | 20 |  | -11.7 | -1.2 | 273 | 281 | 622 | 630 |
| SSU05_0836 | hypothetical protein SSU05_0836 | -12.4 | 0.006 | 81 | 92 | -80 | -69 |  | -8.59 | 0.09 | 82 | 91 | -79 | -70 |
| SSU05_0308 | hypothetical protein SSU05_0308 | -12.3 | 0.006 | 138 | 154 | -80 | -67 |  | -12.6 | -1.58 | 254 | 263 | 123 | 132 |
| SSU05_1886 | Ketol-acid reductoisomerase | -12.2 | 0.007 | 105 | 120 | 4 | 19 |  | -7.56 | 0.51 | 253 | 268 | -167 | -151 |
| SSU05_2060 | 3-hexulose-6-phosphate synthase and related proteins | -12 | 0.007 | 36 | 50 | -1 | 15 |  | -8.97 | -0.07 | 255 | 264 | 369 | 379 |
| SSU05_2047 | 2,3,4,5-tetrahydropyridine-2-carboxylate N-succinyltransferase, putative | -11.8 | 0.008 | 132 | 145 | -3 | 11 |  | -6.92 | 0.78 | 105 | 118 | -147 | -134 |
| SSU05_1656 | Exonuclease VII, large subunit | -11.6 | 0.01 | 2 | 14 | -77 | -65 |  | -11.3 | -1.05 | 3 | 13 | -75 | -65 |
| SSU05_0270 | conserved hypothetical protein | -11.5 | 0.01 | 146 | 158 | -71 | -58 |  | -9.52 | -0.3 | 72 | 89 | 686 | 704 |
| SSU05_0728 | Oligoendopeptidase F | -11.4 | 0.011 | 100 | 116 | 2 | 18 |  | -10.2 | -0.56 | 267 | 292 | 1627 | 1651 |
| SSU05_1791 | Integrase | -11.3 | 0.011 | 81 | 91 | -44 | -34 |  | -10.5 | -0.72 | 73 | 89 | -42 | -30 |
| SSU05_1923 | Uncharacterized protein conserved in bacteria | -11.2 | 0.012 | 120 | 134 | 3 | 17 |  | -6.58 | 0.92 | 246 | 263 | 165 | 180 |
| SSU05_2133 | ABC transporter substrate-binding protein - maltose/maltodextrin | -11.2 | 0.013 | 34 | 48 | 1 | 19 |  | -5.85 | 1.22 | 123 | 131 | 416 | 424 |
| SSU05_1290 | Glycosyltransferase | -11.1 | 0.013 | 112 | 123 | -78 | -67 |  | -11.3 | -1.05 | 246 | 263 | 567 | 582 |
| SSU05_0671 | Exonuclease III | -11 | 0.014 | 12 | 21 | -65 | -56 |  | -10.1 | -0.52 | 185 | 192 | 21 | 28 |
| SSU05_0468 | Predicted membrane GTPase involved in stress response | -10.9 | 0.014 | 83 | 93 | -55 | -45 |  | -9.44 | -0.27 | 138 | 151 | -90 | -73 |
| SSU05_0394 | Glucan phosphorylase | -10.9 | 0.014 | 84 | 101 | -80 | -62 |  | -8.75 | 0.02 | 148 | 161 | 370 | 383 |
| SSU05_0855 | Short-chain alcohol dehydrogenase of unknown specificity | -10.9 | 0.015 | 41 | 53 | -1 | 12 |  | -11.1 | -0.94 | 107 | 127 | 34 | 53 |
| SSU05_1650 | Uncharacterized protein conserved in bacteria | -10.5 | 0.018 | 37 | 51 | -9 | 6 |  | -8.25 | 0.23 | 246 | 263 | 355 | 373 |
| SSU05_1822 | Uncharacterized protein conserved in bacteria | -10.3 | 0.02 | 107 | 122 | -70 | -55 |  | -7.76 | 0.43 | 255 | 266 | -152 | -142 |
| SSU05_1316 | tRNA delta (2)-isopentenylpyrophosphate transferase | -9.92 | 0.024 | 95 | 104 | -74 | -65 |  | -10.8 | -0.84 | 254 | 263 | 563 | 572 |
| SSU05_1703 | CBS domain protein | -9.83 | 0.025 | 79 | 92 | -40 | -27 |  | -8.21 | 0.25 | 91 | 114 | -185 | -160 |
| SSU05_0692 | Pyruvate/2-oxoglutarate dehydrogenase complex, dihydrolipoamide dehydrogenase (E3) component, and related enzymes | -9.79 | 0.026 | 51 | 69 | 1 | 19 |  | -10.5 | -0.69 | 83 | 108 | 376 | 404 |
| SSU05_0426 | tRNA and rRNA cytosine-C5-methylase | -9.66 | 0.028 | 120 | 130 | -19 | -9 |  | -9.34 | -0.22 | 262 | 273 | 619 | 630 |
| SSU05_0684 | type III restriction-modification system, restriction endonuclease subunit | -9.49 | 0.03 | 39 | 51 | -2 | 11 |  | -7.09 | 0.71 | 267 | 280 | 2964 | 2976 |
| SSU05_2015 | RNase P protein component | -9.33 | 0.032 | 94 | 108 | -69 | -55 |  | -6.62 | 0.9 | 152 | 161 | 374 | 383 |
| SSU05_0583 | Transposase and inactivated derivative | -9.29 | 0.033 | 12 | 20 | -59 | -51 |  | -6.68 | 0.88 | 171 | 178 | -150 | -143 |
| SSU05_0184 | Beta-glucosidase/6-phospho-beta-glucosidase/beta- galactosidase | -9.19 | 0.034 | 129 | 141 | -65 | -53 |  | -6.39 | 1 | 92 | 103 | 588 | 599 |
| SSU05_1758 | hypothetical protein SSU05_1758 | -9.16 | 0.035 | 94 | 105 | -73 | -62 |  | -9.87 | -0.44 | 13 | 22 | -180 | -171 |
| SSU05_1383 | Peroxiredoxin | -9.08 | 0.036 | 141 | 154 | -79 | -66 |  | -7.73 | 0.44 | 96 | 110 | -123 | -106 |
| SSU05_0492 | ATPases with chaperone activity, ATP-binding subunit | -9.04 | 0.037 | 145 | 155 | 1 | 11 |  | -13.6 | -1.98 | 146 | 159 | 700 | 714 |
| SSU05_1211 | hypothetical protein SSU05_1211 | -8.93 | 0.038 | 8 | 17 | -71 | -62 |  | -10.5 | -0.69 | 183 | 194 | -21 | -10 |
| SSU05_1582 | hypothetical protein SSU05_1582 | -8.85 | 0.04 | 132 | 148 | -35 | -20 |  | -8.76 | 0.02 | 251 | 263 | -99 | -86 |
| SSU05_1762 | hypothetical protein SSU05_1762 | -8.65 | 0.043 | 95 | 105 | -80 | -70 |  | -8.79 | 0 | 96 | 104 | -78 | -70 |
| SSU05_1402 | putative N-acetylmannosamine 6-P epimerase | -8.63 | 0.043 | 102 | 116 | -48 | -34 |  | -11 | -0.93 | 100 | 115 | -46 | -31 |
| SSU05_1974 | transcriptional regulator | -8.61 | 0.044 | 117 | 132 | 5 | 19 |  | -4.51 | 1.78 | 257 | 274 | -146 | -126 |
| SSU05_0489 | Isoleucyl-tRNA synthetase | -8.59 | 0.044 | 42 | 55 | -3 | 11 |  | -14.5 | -2.38 | 174 | 197 | 525 | 547 |
| SSU05_0194 | Uncharacterized protein conserved in bacteria | -8.49 | 0.046 | 40 | 55 | -3 | 12 |  | -6.65 | 0.89 | 85 | 93 | 22 | 30 |
| SSU05_2129 | Permease of the major facilitator superfamily | -8.33 | 0.049 | 7 | 22 | -77 | -62 |  | -5.1 | 1.54 | 246 | 261 | -199 | -184 |
| SSU05_0284 | ABC-type multidrug transport system, ATPase and permease component | -8.31 | 0.05 | 41 | 51 | -69 | -59 |  | -5.79 | 1.25 | 249 | 277 | 521 | 545 |
| SSU05_1987 | Pseudouridylate synthase, 23S RNA-specific | -8.31 | 0.05 | 107 | 119 | -71 | -59 |  | -7.1 | 0.71 | 267 | 281 | 820 | 834 |

TargetRNA2 and RNAPredator tools were used to predict sRNA23 target genes. For TargetRNA2 tool; Target parameters: NTs 80 and 20 from the start codon upstream and downstream, respectively. RNAPredator targets on the genes from transcription start site to transcription terminate site. The corresponding *P* < 0.05, energy < −10 kcal/mol with TargetRNA2 marked in red text were taken as the threshold to consider an interaction as positive (n=25). The genes shown in the table highlighted in green color indicate the overlapped target genes predicted from both tools (TargetRNA2 and RNAPredator) were also considered as interaction positive (n=6).

| **Table S4. List of the proteins with RNA-binding identified by mass spectrum** | | | | | |
| --- | --- | --- | --- | --- | --- |
| Protein  _name | Description | Mw  (kDa) | PI | Gene_id in NZ_CP020863 | Gene_id in CP000407 |
| FBA | fructose-1,6-bisphosphate aldolase | 31.15 | 4.9 | B9H01_01725 | SSU05_0336 |
|  |  |  |  |  | SSU05_0337 |
|  |  |  |  |  | SSU05_0338 |
|  |  |  |  |  | SSU05_0339 |
| rplB | 50S ribosomal protein L2 | 29.86 | 10.7 | B9H01_00460 | SSU05_0074 |
| accD | acetyl-CoA carboxylase carboxyl transferase subunit beta | 31.83 | 6.97 | B9H01_08645 | SSU05_1797 |
|  |  |  |  |  | SSU05_1798 |

| **Table S5. Bacterial strains, plasmids and primers** | | |
| --- | --- | --- |
| **Bacterial strains** | | |
| **Strains** | **Description** | **Source** |
| *S. suis* 05ZYH33 | *S. suis* serotype 2, wild type | Store in our lab |
| *S. suis* SC19 | *S. suis serotype* 2, wild type | Store in our lab |
| *E. coli* top 10 | Host strain for plasmid amplification | Transgen biotech |
| *E. coli* BL21 (DE3) | Expression host strain | Store in our lab |
| ∆sRNA23 | The deletion strain of sRNA34, Spc^R^ | This study |
| C-∆sRNA23 | Complete to ∆sRNA23, Spc^R^ | This sudy |
| ∆sRNA24 | The deletion strain of sRNA24, Spc^R^ | This sudy |
| ∆sRNA76 | The deletion strain of sRNA76, Spc^R^ | This sudy |
| **Plasmids** | | |
| **Plasmids** | **Description** | **Source** |
| pEASY-T1 | TA clone | Transgen biotech |
| pSET4S | Suiside plasmid | Store in our lab |
| pSET2 | Shuttle plasmid with adding a teminator, Spc^R^ | Store in our lab |
| pSET4S-sRNA23 | Homologous recombination plasmid for ∆sRNA23, Spc^R^ | This study |
| pSET4S-sRNA24 | Homologous recombination plasmid for ∆sRNA24, Spc^R^ | This study |
| pSET4S-sRNA76 | Homologous recombination plasmid for ∆sRNA76, Spc^R^ | This study |
| pSET2-CsRNA23 | Shuttle complementation plasmid of sRNA23, Spc^R^ | This study |
| pET28a (+) | Expression vector with hexahistidine tag, Kan^R^ | Novagen |
| pET28a-fbpa | Expression vector for protein fba | This study |
| pET28a-*accD* | Expression vector for protein accD | This study |
| pET28a-*rplB* | Expression vector for protein rplB | This study |
| **primers** | | |
| **Primers** | **sequence (5'-3')** | **Amplification gene** |
| **For RT-PCR identification of predicted sRNAs** | | |
| g-gyrA-F | ATACAGAAGCACGTATGAGCAA | gyrA |
| g-gyrA-R | TTGTGTGGAGGAATGTTGGTAG |  |
| q-sRNA76-F | TGATGTGTTGAAATAAGCGATGT | sRNA76 |
| q-sRNA76-R | AAACCAGACCAACCTACCAAG |  |
| q-sRNA23-F | AAATTTAGAGCCTGTCAACCCA | sRNA23 |
| q-sRNA23-R | CAGTTAGTCCCACAGAACAACA |  |
| q-sRNA24-F | AGGTGATGCCAAAGTGTATAGG | sRNA24 |
| q-sRNA24-R | ACTTAACACCATCCAACTCCAC |  |
| q-sRNA14-F | ACAGTTTGAGGTTGGAGATGAA | sRNA14 |
| q-sRNA14-R | GGTTCTAGTGCAAGTACACTTCT |  |
| q-sRNA31-F | TTCACTGTTCAGCACTTCTATACTA | sRNA31 |
| q-sRNA31-R | GTATCAAACCTGTATTCACAAGCG |  |
| q-sRNA19-F | TTACACTCCACATCTTCCTCTAC | sRNA19 |
| q-sRNA19-R | TCGCTACAGAACAGTCAGAATAG |  |
| q-sRNA40-F | TTAAGTCCAACCCTTACCCTTG | sRNA40 |
| q-sRNA40-R | ATCCGACGACTAGCCCAAA |  |
| q-sRNA28-F | GACAAGTTGAGTTCTTTCCCAC | sRNA28 |
| q-sRNA28-R | TCGTTTCCGATGAGAATAGAAGG |  |
| q-sRNA16-F | TCCAAACTAGCTGTTAGCGATG | sRNA16 |
| q-sRNA16-R | CTATTGGTCCTCTTGCGCC |  |
| q-sRNA11-F | CATCGCAACAACCGTTACATT | sRNA11 |
| q-sRNA11-R | GAAAGTGGAAATAGTACAGATGGG |  |
| **Primers for Northern blot** | | |
| sRNA23-probe-F | ACGTTTCCGATAGGAGGAAGC | sRNA23 |
| sRNA23-probe-R | TTTTTGTACAGAAGAAGCCATTGG |  |
| **Primers for RACE** | | |
| Long primer | CTAATACGACTCACTATAGGGCAAGCAGTGGTATCAACGCAGAGT |  |
| Short primer | CTAATACGACTCACTATAGGGC |  |
| 5**'**RACE-GSP | GATTACGCCAAGCTTTTGGAGGCACAGTTGGGTTGACA |  |
| 3**'**RACE-GSP | GTCATCAGTTGGCTTAGTACAGCGTGG |  |
| **Primers for strain construction** | | |
| M13F | TGTAAAACGACGGCCAGT |  |
| sRN23-LA-F | AAAACGACGGCCAGTGAATTCCGTGAGTCGGATTTAGACTTTTCG (*EcoR* I) | The left arm of the sRNA23 |
| sRN23-LA-R | CTAACCTCCTATCGGAAACGTAGTAACA |  |
| sRN23-RA-F | CGTTTCCGATAGGAGGTTAGTTAATTAATTTGCAAATCCTTCATA | The right arm of sRNA23 |
| sRN23-RA-R | CAGGTCGACTCTAGAGGATCCTCCAAAAAATAAAAATATTAATATAAAAACTAGG (*BamH* I) |  |
| sRN23-In-R | TGAGCCCAGTTAGTCCCAC | Inner region of sRNA23 |
| sRN23-Out-F | GATAAGTGAAATGGGCGAAA | Outer region of sRNA23 |
| sRN23-Out-R | CACTTTGGCATCACCTCG |  |
| sRN24-LA-F | AAAACGACGGCCAGTGAATTCATTTGAATGGATTATATATAACATTAAGTTGG (*EcoR* I) | The left arm of the sRNA24 |
| sRN24-LA-R | TGACTTCGTCTCTACAAATGATAGTATAGCACAA |  |
| sRN24-RA-F | CATTTGTAGAGACGAAGTCAATTAAATTTGCAAATCCTTCA | The right arm of sRNA24 |
| sRN24-RA-R | CAGGTCGACTCTAGAGGATCCAATCGCTTTTTGAAGCTGCTCA (*BamH* I) |  |
| sRN24-In-R | GTCATTAGCAAGACCAAAGC | Inner region of sRNA24 |
| sRN24-Out-F | GTTCTGTGGGACTAACTGGG | Outer region of sRNA24 |
| sRN24-Out-R | ATCAACAGAGCAAGAGCCA |  |
| sRN76-LA-F | AAAACGACGGCCAGTGAATTCTTTAGTAAGAACAGATATACTTCCGATAGTG (*EcoR* I) | The left arm of the sRNA76 |
| sRN76-LA-R | TTACAGCGGGGTATAGGGTTTGCCAGCAATTTG |  |
| sRN76-RA-F | AACCCTATACCCCGCTGTAAACCAAGATCATC | The right arm of sRNA76 |
| sRN76-RA-R | CAGGTCGACTCTAGAGGATCCACGTGATTTGCTGATTCCAGC (*BamH* I) |  |
| sRN76-In-R | CATTGGTGCTCTGCGAAA | Inner region of sRNA76 |
| sRN76-Out-F | TGCTGACATATTTCCTCCTA | Outer region of sRNA76 |
| sRN76-Out-R | GTCGAACGGACTTGCTAT |  |
| pSET2-CsRNA23-F | TGAGCGCAACGCAATCTCGAGCGGAAATGAGGTGTTTTTAGTTGA (*Xho* I) | Construction complementary vector of sRNA23 |
| pSET2-CsRNA23-R | AGAATAGGCGCGCCTGAATTCTTTTTGTACAGAAGAAGCCATTGG (*EcoR* I) |  |
| pET28a-rplB-F | GTTTAACTTTAAGAAGGAGATATACCATGGCGCATCATCATCATCATCACGTGGGTATTAAAGTTTATAAACC (*Nco* I) | rplB |
| pET28a-rplB-R | GCCGCAAGCTTGTCGACGGAGCTCGAATTCTTATTTTTGGTTGCGACGACGAACGATAAGTTTGTCAG (*EcoR* I) |  |
| pET28a-accD-F | GTTTAACTTTAAGAAGGAGATATACCATGGCGCATCATCATCATCATCACATGGCTTTGTTTCGCAAA (*Nco* I) | accD |
| pET28a-accD-R | GCCGCAAGCTTGTCGACGGAGCTCGAATTCTCATCTGACACCTCCATGCATTCTCAGCAAGCGACTGA (*EcoR* I) |  |
| pET28a-fba-F | GTTTAACTTTAAGAAGGAGATATACCATGGCGCATCATCATCATCATCACATGCCATTAGTTTCAGCA (*Nco* I) | fba |
| pET28a-fba-R | GCCGCAAGCTTGTCGACGGAGCTCGAATTCTTAAGCTTTGTTCGCTGAACCGAATACGTCGA (*EcoR* I) |  |
| **Primers for RNA pull-down** | | |
| sRNA23-Sense-F | GATCACTAATACGACTCACTATAGGGACGTTTCCGATAGGAGGAAG (T7 Promoter) | In vitro transcripion template for sRNA23-Sense |
| sRNA23-Sense-R | TTTTTGTACAGAAGAAGCCATTG |  |
| sRNA23-Antisense-F | GATCACTAATACGACTCACTATAGGGTTTTTGTACAGAAGAAGCCATTG (T7 Promoter) | In vitro transcripion template for sRNA23-Antisense |
| sRNA23-Antisense-R | ACGTTTCCGATAGGAGGAAG |  |
| **Primers for predicted genes interacting with sRNA23** | | |
| q-SSU05_1689-F | CAGCCAGATAGCGGAACAC | SSU05_1689 |
| q-SSU05_1689-R | GGATGAGATGAGTGAGCGTTT |  |
| q-SSU05_1473-F | AGCTCCTTGAGGTGATTGGG | SSU05_1473 |
| q-SSU05_1473-R | TAGCAGCCAGAACAGACAGG |  |
| q-SSU05_1922-F | GTATCTGGCGCAAACAGACC | SSU05_1922 |
| q-SSU05_1922-R | CAATCGAAGTGGCGAAACGA |  |
| q-SSU05_0048-F | CCTGATGTTGCTTATAGCGGG | SSU05_0048 |
| q-SSU05_0048-R | GACACTGCATGAAACTCCGT |  |
| q-SSU05_2181-F | TCCATTCAGGAGGTGCCATT | SSU05_2181 |
| q-SSU05_2181-R | AGCTTGATCCTCTTGGTGCT |  |
| q-SSU05_0166-F | GCACACGGAGTTGGTCTATG | SSU05_0166 |
| q-SSU05_0166-R | ATTCGTCAATCACCCGACCA |  |
| q-SSU05_0024-F | AGAGCGTGTCAGTATCGTCC | SSU05_0024 |
| q-SSU05_0024-R | CATCTGCCTGACCATCCTTG |  |
| q-SSU05_0308-F | AGTGTCTGGCTCATTCTGCT | SSU05_0308 |
| q-SSU05_0308-R | AAATTGCCCAGCAGTCAGG |  |
| q-SSU05_1886-F | GTGCGTGCAGGTAAGTCATT | SSU05_1886 |
| q-SSU05_1886-R | ACGTTGAAACCATGGGCAAA |  |
| q-SSU05_2060-F | TCCCAAGCGATTTACCACCA | SSU05_2060 |
| q-SSU05_2060-R | CTTCTGTGATACCGCGACCT |  |
| q-SSU05_2047-F | TATGTGGTGGAGCAAGACGG | SSU05_2047 |
| q-SSU05_2047-R | CGATTTCCGCACCGATGTT |  |
| q-SSU05_1656-F | CAAGCTCTGACACAGGAAGG | SSU05_1656 |
| q-SSU05_1656-R | TTGCACCTTGGTCGGATAGA |  |
| q-SSU05_0270-F | CAAGATAGCACCGACCGAAC | SSU05_0270 |
| q-SSU05_0270-R | CTGACACGTTTGTTGGGCTT |  |
| q-SSU05_0728-F | CAACGCATCACACCTGAAGA | SSU05_0728 |
| q-SSU05_0728-R | AGAGTAGCACAGAAGCCACC |  |
| q-SSU05_1791-F | GCAAATCTTCCTGTCGTCACC | SSU05_1791 |
| q-SSU05_1791-R | TTCTGGGTCGTTGTTATGCTTG |  |
| q-SSU05_1923-F | TAGCCATACCAAGGCCAAGA | SSU05_1923 |
| q-SSU05_1923-R | CACCAACCTACCAGTTCATCC |  |
| q-SSU05_2133-F | CAGCATTCGGTGGTGGTAAA | SSU05_2133 |
| q-SSU05_2133-R | GAACTTCGTTTGTAGCGTCGT |  |
| q-SSU05_1290-F | ACAGCAGATAGAGCGACCAA | SSU05_1290 |
| q-SSU05_1290-R | CACTTGGCCCGTTTCCATTC |  |
| q-SSU05_0671-F | ACGCCAAATGCTGGTGATG | SSU05_0671 |
| q-SSU05_0671-R | AGATTGACGGTTACTTGCGG |  |
| q-SSU05_0468-F | CAGATGCAGTTGAGGCACTT | SSU05_0468 |
| q-SSU05_0468-R | AGGGTCAACACGGAGAGAAA |  |
| q-SSU05_0394-F | GGTATCACTTTCCGCCGTTG | SSU05_0394 |
| q-SSU05_0394-R | GTCATCAGCAAAGGCAAGCA |  |
| q-SSU05_0855-F | ACCATGTTCAGGTGTCAGTTC | SSU05_0855 |
| q-SSU05_0855-R | TTGTCCAATCCCAATGCCAG |  |
| q-SSU05_1650-F | GTCAAGGGTGGTCGTATTGG | SSU05_1650 |
| q-SSU05_1650-R | AGGTCTTATTGCCTCGTCCC |  |
| q-SSU05_1822-F | GCGAGATTGTTATCGCACCT | SSU05_1822 |
| q-SSU05_1822-R | CGACCTTCCTCGTCAGTTTC |  |
| q-SSU05_1762-F | GTGTATGCCTTGTTGGTGCT | SSU05_1762 |
| q-SSU05_1762-R | CAGGAGAAGCTTTCACTTCCTT |  |
| q-SSU05_1402-F | TCGGGCCAATAGTGTACGAG | SSU05_1402 |
| q-SSU05_1402-R | TCGTCTACTTCTCGCATCGT |  |
| g-16s rRNA-F | GGTAATAAACCGGAGGAAGGTG | 16S rRNA |
| g-16s rRNA-R | CTACAATCCGAACTGAGACTGG |  |
