# Supplementary figures and images for: sRNA23, a novel small RNA, regulates to the pathogenesis of Streptococcus suis serotype 2
Source: Virulence. 2021 Dec 9;12(1):3045–61. doi: 10.1080/21505594.2021.2008177 (PMC8667912; doi:10.1080/21505594.2021.2008177)

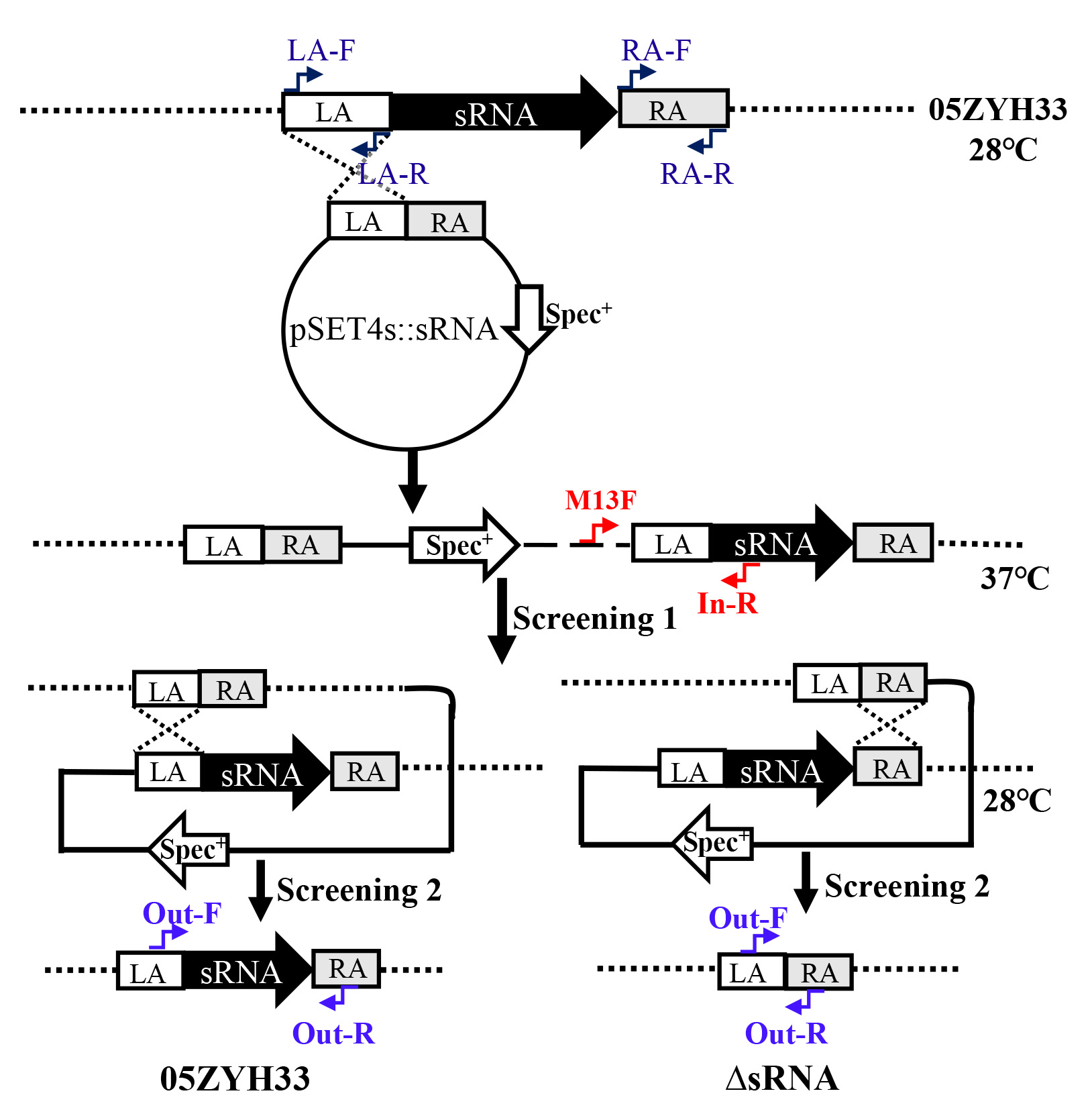

Supplement: Supplemental Material [file KVIR_A_2008177_SM9937.zip › supplementary/Figure_S1.jpg]

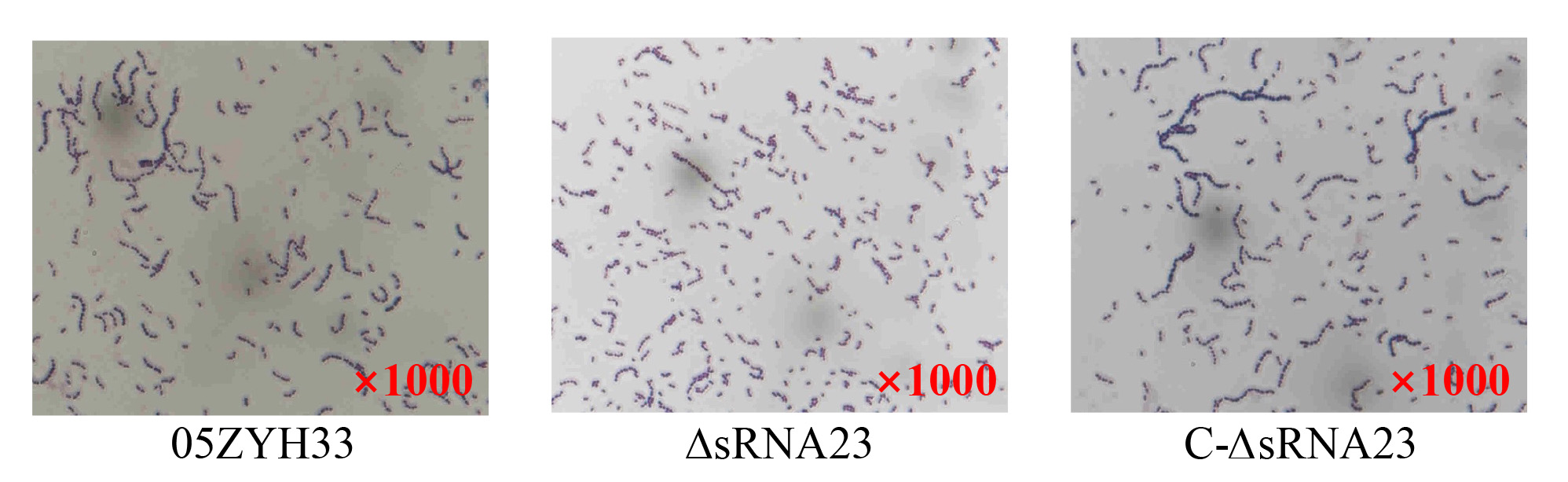

Supplement: Supplemental Material [file KVIR_A_2008177_SM9937.zip › supplementary/Figure_S2.jpg]

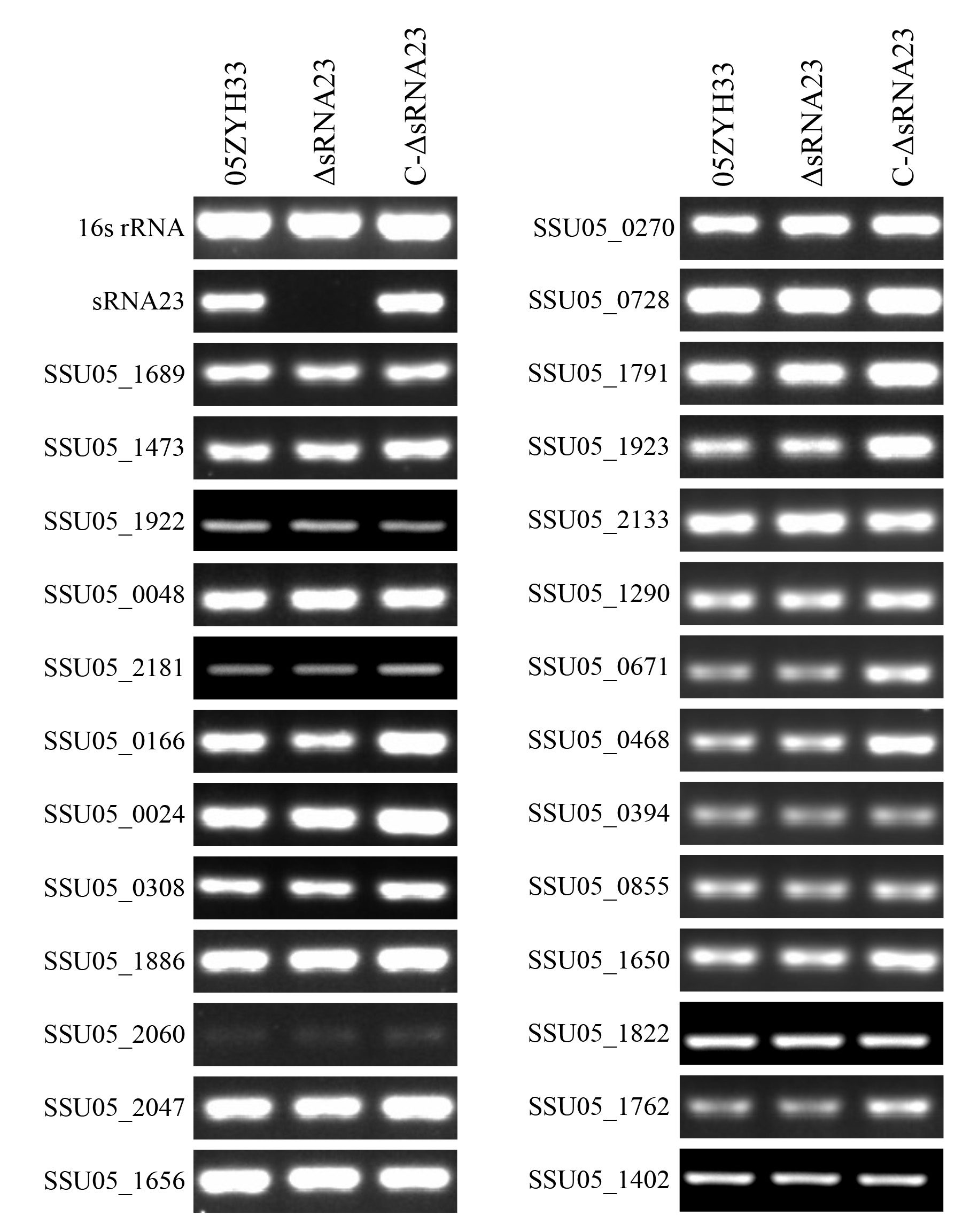

Supplement: Supplemental Material [file KVIR_A_2008177_SM9937.zip › supplementary/Figure_S3.jpg]
